# Draft Genome Sequence of *Klebsiella pneumoniae* 704SK6, an OXA-48- and CTX-M-15-Encoding Wastewater Isolate

**DOI:** 10.1128/genomeA.00831-17

**Published:** 2017-08-17

**Authors:** Roger Marti, Roger Stephan, Jochen Klumpp, Magdalena Nüesch-Inderbinen, Jörg Hummerjohann, Claudia Bagutti, Katrin Zurfluh

**Affiliations:** a Institute for Food Safety and Hygiene, Vetsuisse Faculty University of Zurich, Zurich, Switzerland; b Institute of Food, Nutrition and Health, ETH Zurich, Zurich, Switzerland; c Division of Food Microbial Systems, Microbiological Safety of Foods of Animal Origin Group, Agroscope, Bern, Switzerland; d Biosafety Laboratory, State Laboratory Basel-City, Basel, Switzerland

## Abstract

The Swiss wastewater isolate *Klebsiella pneumoniae* 704SK6, encoding OXA-48 and CTX-M-15 β-lactamases, was fully sequenced. The assembly resulted in an open chromosome of 5,208,104 bp in size (G+C content, 57.6%) and four closed plasmid sequences of 209,651, 197,670, 65,998, and 63,605 bp in size.

## GENOME ANNOUNCEMENT

*Klebsiella pneumoniae* is pervasive in the environment and an intestinal commensal. It is also a severe threat to human health, as it causes both nosocomial and community-acquired infections (1). OXA-48 is a widespread class D β-lactamase which is not susceptible to β-lactamase inhibitors. While it does not hydrolyze extended-spectrum cephalosporins, it degrades penicillins very efficiently and carbapenems at lower, but still clinically significant, rates (2). OXA-48 producers frequently also encode extended-spectrum β-lactamases (ESBLs), resulting in complete β-lactam resistance and treatment failure with this class of antibiotics (3).

*K. pneumoniae* isolate 704SK6 was isolated from wastewater near Basel, Switzerland, in December 2015 (4). The genome was sequenced at the Functional Genomics Center Zurich (FGCZ) using Pacific Biosciences (PacBio) single-molecule real-time (SMRT) technology RS2 reads (C4/P6 chemistry). *De novo* assembly was carried out using SMRT Analysis 2.3 with the HGAP3 protocol, and sequences were annotated using the NCBI Prokaryotic Genome Annotation Pipeline (5). Sequence type (ST), acquired antibiotic resistances, and plasmid incompatibility (Inc) groups were assessed using the MLST-1.8 server (6), ResFinder 2.1 (7), and PlasmidFinder 1.3 (8), respectively (see http://www.genomicepidemiology.org/).

The chromosome of *K. pneumoniae* 704SK6 (ST437) is not fully closed. The linear sequence is 5,208,104 bp in length, with a G+C content of 57.6%, and it contains β-lactam (*bla*_SHV-11_), fosfomycin (*fosA*), and quinolone (*oqxA* and *oqxB*) resistance genes. Assembly revealed four complete (closed) plasmid sequences, which are provided here in descending order of size: (i) p704SK6_1 (209,651 bp; G+C content, 45.2%) carries no acquired resistance genes but otherwise shows high similarity to the *bla*_VIM_-carrying *K. pneumoniae* plasmid pKP04VIM (GenBank accession no. KU318421, 91% query coverage, 99% identity); (ii) p704SK6_2 (197,670 bp; G+C content, 52.9%; IncFII) contains aminoglycoside [*aph*(*3′*)-*Ia*], macrolide [*mph*(A)], and sulfonamide (*sul1*) resistance genes and is very similar to *K. pneumoniae* plasmid p34618 (accession no. CP010393, 98% query coverage, 99% identity); (iii) p704SK6_3 (65,998 bp; G+C content, 51.0%; IncFIB) features a class 1 integron (In1407 [9]) and is a multidrug resistance (MDR) plasmid carrying aminoglycoside [*aac*(*3*)*-IInd*], β-lactam (*bla*_OXA-1_, *bla*_TEM-1B_, and *bla*_CTX-M-15_), fluoroquinolone/aminoglycoside [*aac*(*6′*)*-Ib-cr*], tetracycline (*tetD*), and trimethoprim (*dfrA30*) resistance genes, and it most closely resembles *K. pneumoniae* plasmid p_IncFIB_DHQP1002001 (accession no. CP016810, 67% query coverage, 99% identity); (iv) p704SK6_4 (63,605 bp; G+C content, 51.2%; IncL) encodes the OXA-48 carbapenemase and is very similar to pOXA-48 (accession no. JN626286, 95.1% identical on nucleotide level). There are some notable differences between these two plasmids; the gene encoding OXA-48 lies between two IS*1999* transposases and is 100% identical in the two plasmids, but it is located on opposite strands. Also, our newly sequenced plasmid features a 504-bp insertion element, IS*1* protein InsB (accession no. WP_001119291), directly upstream (complementary strand) of its OXA-48 gene, as well as downstream (complementary strand) of its *korC* gene as part of a 776-bp insertion. Excluding these regions, the two plasmids are 99.4% identical.

Isolates like *K. pneumoniae* 704SK6 highlight the wide dissemination of pan-β-lactam-resistant strains of this species and the only slightly modified, ever-reoccurring, pOXA-48 IncL plasmid.

### Accession number(s).

Sequence and annotation data of the genome have been deposited in GenBank under accession numbers CP022143 (chromosome), CP022144 (p704SK6_1), CP022145 (p704SK6_2), CP022146 (p704SK6_3), and CP022147 (p704SK6_4). This is the first version of this genome.
